# Cesium and Strontium Retentions Governed by Aluminosilicate Gel in Alkali-Activated Cements

**DOI:** 10.3390/ma10040447

**Published:** 2017-04-23

**Authors:** Jeong Gook Jang, Sol Moi Park, Haeng Ki Lee

**Affiliations:** 1 Korea Institute of Geoscience and Mineral Resources, 124 Gwahak-ro, Yuseong-gu, Daejeon 34132, Korea; jangjg@kigam.re.kr; 2 Department of Civil and Environmental Engineering, Korea Advanced Institute of Science and Technology, 291 Daehak-ro, Yuseong-gu, Daejeon 34141, Korea; solmoi.park@kaist.ac.kr

**Keywords:** alkali-activated cements, gel, cesium, strontium, adsorption kinetics, immobilization

## Abstract

The present study investigates the retention mechanisms of cesium and strontium for alkali-activated cements. Retention mechanisms such as adsorption and precipitation were examined in light of chemical interactions. Batch adsorption experiments and multi-technical characterizations by using X-ray diffraction, zeta potential measurements, and the N_2_ gas adsorption/desorption methods were conducted for this purpose. Strontium was found to crystalize in alkali-activated cements, while no cesium-bearing crystalline phases were detected. The adsorption kinetics of alkali-activated cements having relatively high adsorption capacities were compatible with pseudo-second-order kinetic model, thereby suggesting that it is governed by complex multistep adsorption. The results provide new insight, demonstrating that characteristics of aluminosilicate gel with a highly negatively charged surface and high micropore surface area facilitated more effective immobilization of cesium and strontium in comparison with calcium silicate hydrates.

## 1. Introduction

Cementitious materials used as a solidifying agent for radioactive waste plays an important role to isolate radionuclides from the external environment by two main functions [1]. The first function is a physical barrier by which the discontinuous internal structure of a cementitious material acts to retard the release of radionuclides and prevent groundwater from infiltrating into the solidified matrix or disposal system, while the second function is a chemical barrier in which radionuclides are chemically immobilized by the chemical elements in a cementitious material [1]. The function of chemical barrier retards the radionuclides that leach into the external environment even when that of a physical barrier has diminished [1]. It is generally accepted that the performance of a cementitious material as a chemical barrier is more important than the performance as a physical barrier in light of the long-term safety of radioactive waste management [2,3].

Cementitious materials currently being used or in the planning phase are Portland cements and blended cements incorporating supplementary cementitious materials such as silica fume, fly ash and blast furnace slag [4]. While the micro-pore structure and the high pH pore solution in hardened Portland cement composites are beneficial for the immobilization of radionuclides [2], the calcium silicate hydrates of Portland cement inevitably inherit weaknesses such as low resistance to acid and fire, and vulnerability to chemical decomposition due to the many salts in groundwater [5]. Moreover, calcium leaching for the long-term increases concern over the matrices being weakened [6,7]. The long-term durability concerns along with high diffusivity of cesium and strontium in a Portland cement matrix have called for the need for development of an alternative binder guaranteeing structural security over an extended period of time and with improved immobilization performance [3,4,8,9].

Alkali-activated cements, which include an alkali-activated slag and a geopolymer, provide comparable performance to that of Portland cements in a range of applications. The application of alkali-activated cements as a solidifying agent for waste-form can offer the following aspects in comparison with Portland cement-based materials: outstanding mechanical properties, exceptional durability over the long-term, excellent thermal resistance, and environmental friendliness [10,11,12,13,14,15,16,17,18,19]. Initial works on the application of alkali-activated cements for the immobilization of radioactive waste reported promising results [20,21]. Further research has thereafter pioneered to correlate the characteristics of alkali-activated cements with its performance in the immobilization of radionuclides [22,23,24,25]. Previous studies focusing on the leaching behavior of various cationic contaminants from alkali-activated cements based on fly ash or metakaolin validated the effectiveness of alkali-activation to immobilize various radionuclides such as Ba, Sr, As and Se [23]. Shi and Fernández-Jiménez [25] reported that the immobilization mechanism of alkali metals such as Li, Na, K, Rb and Cs is that these metals balance the charge in the alkali-activated cement. Similarly, a recent study conducted by the authors showed that the diffusivity of cesium and strontium was significantly lower in alkali-activated fly ash-based waste forms than that in a Portland cement-based waste form [24]. However, previous studies are particularly lacking in a clear understanding of the retention mechanism of alkali-activated cements, especially in chemical aspects.

This study aims to elucidate the retention mechanisms of cesium and strontium in alkali-activated cements. Alkali-activated fly ash and alkali-activated fly ash/slag blends are investigated as binder materials. Electrochemical adsorption by the surface of hydrates, incorporation in an amorphous gel, and precipitation and crystallization as a solid phase were examined in light of chemical interactions. Batch adsorption experiments and multi-technical characterizations by using X-ray diffraction (XRD), zeta potential measurements, and the N_2_ gas adsorption/desorption methods were conducted for this purpose.

## 2. Materials and Methods

### 2.1. Materials and Sample Preparation

Fly ash and ground granulated blast furnace slag were used for synthesis of binders. Ordinary Portland cement was used as a reference material. Chemical compositions of the raw materials are summarized in Table 1. To produce alkali-activators, a sodium hydroxide solution and sodium silicate solution with SiO_2_ = 29 wt %, Na_2_O = 10 wt % and H_2_O = 61 wt % were used.

Four types of alkali-activated cements were investigated as a binder. The mix design of the samples is summarized in Table 2. A reference material, Portland cement (PC) paste, was produced from a mixture with a water to cement ratio of 0.5. A fly ash-based binder (S0) was produced from a mixture with liquid (alkali-activator) to solid (fly ash) ratio of 0.5. A mixture of a 9 M NaOH solution and a sodium silicate solution at a weight ratio of 1:1 was used as alkali-activator. Fly ash/slag blended binder (S1, S3 and S5) were produced by the replacement of fly ash with slag at 10%, 30% and 50% by weight. The fly ash/slag blended binders were produced from mixtures with liquid (alkali-activator) to solid (fly ash and slag) ratio of 0.5, identical to that of the other matrices. The alkali-activator used for the fly ash/slag blended binder was composed with a mixture of a 4 M NaOH solution and a sodium silicate solution at a weight ratio of 2:1. The activators used in this study were determined from a former work conducted by the authors, considering reaction degree and mechanical properties of the materials [24].

To investigate the interaction between binder matrix and cesium/strontium, test samples incorporating cesium or strontium were prepared. These samples were used for tests of microscopic observation, X-ray diffraction analysis, and zeta potential measurement. Analytical reagent grade CsCl and SrCl_2_∙6H_2_O (both supplied by Samchun Pure Chemical Co., Ltd., Pyeongtaek, Korea) were used to simulate radioactive isotopes. In addition, 12.67 g/L of CsCl and 30.43 g/L of SrCl_2_∙6H_2_O were incorporated into the binder to reach 10 g/L of Cs^+^ and Sr^2+^ to be incorporated relative to the volume of a sample. Cesium chloride and strontium chloride were dissolved into a liquid (water or alkali-activator) before the mixing of the sample.

To make the samples, all ingredients were uniformly mixed for five minutes. The fresh mixture was introduced into a cylindrical vial mold with a diameter of 25 mm and a height of 50 mm. The lid of the vial mold was tightly closed after the fresh mixture was poured. The fly ash-based binder samples were initially cured at 60 °C for 24 h to induce geopolymerization. The fly ash/slag blended binders and the PC samples were initially cured at 20 °C, since the reaction of these materials can occur at ambient temperatures [26,27]. All samples were exposed at 20 °C for a further four weeks to simulate the mature state of a binder matrix.

### 2.2. Test Methods

A microscope image analysis was conducted on the surface of a crushed bulk sample using a UC-CAM digital microscope (Shenzhen Technology, Shenzhen, China)to observe the internal matrix and the precipitates of cesium and strontium. In order to identify the changes of crystalline phases by incorporating cesium and strontium into a solidified matrix, an XRD analysis was conducted using a SmartLab device (Rigaku, Tokyo, Japan), with CuKα radiation at 40 kV and 30 mA. The XRD data were collected in the 2θ scan range of 3° to 60° at a scan speed of 0.2°/min. Batch adsorption experiments were conducted to evaluate the Cs^+^ and Sr^2+^ adsorption behaviors on the binders. Cured binders were ground and sieved to yield a specified particle size smaller than 100 μm. For the adsorption test, 0.5 g of ground binder material was mixed with 500 mL of a 1 g/L Cs^+^ and Sr^2+^ solution in a polypropylene vessel with a volume of 1 L. The vessel was stored at 20 °C (293 K) and was stirred in a shaker for 5 min per day. Water samples were collected at determined time intervals (1, 3, 24, 72, and 120 h). The residual Cs^+^ and Sr^2+^ in the collected solution were quantified by means of inductively coupled plasma mass spectrometry (Bruker-820MS, Bremen, Germany) and inductively coupled plasma optical emission spectrometry (iCAP7400DUO, Thermo scientific, Waltham, MA, USA), respectively. The data obtained from the batch adsorption experiments were used to compute the adsorption capacity *q_t_* (mg/g) using the mass–balance relationship, which represents the amount of adsorbed Cs^+^ or Sr^2+^ per amount of binder [28]:

(1) $$q_{t}=\frac{\left(c_{0}-c_{t}\right)}{m_{s}}V_{s}.$$

Here, *C_0_* and *C_t_* are the concentrations of Cs^+^ or Sr^2+^ in the solution at time *t* = 0 and *t* = t, respectively; *V_s_* is the volume of the solution, and *m_s_* is the mass of the adsorbent [28]. After equilibrium was reached, the distribution factor (*K_d_*) was calculated as follows [29]:

(2) $$K_{d}=\frac{c_{0}-c_{e}}{c_{e}}\frac{V_{s}}{m_{s}},$$

where *C_e_* denotes the equilibrium concentration of Cs^+^ or Sr^2+^ in the solution [29]. Two factors pertaining to the binder, i.e., zeta potential and surface area, which influence the adsorption of cesium and strontium, were characterized. The zeta potential values of the powdered binders were measured using an ELS-Z2 device (Otsuka Electronics, Osaka, Japan). The suspension was prepared to 2.5 g/L in distilled water. The Brunauer–Emmett–Teller (BET) surface area and the Barrett–Joyner–Halenda (BJH) pore size distribution were determined by N_2_ gas adsorption/desorption methods on a Tristar II 3020 instrument (Micromeritics, Norcross, GA, USA). The ground binders used in the two characterization tests were prepared with a specified particle size smaller than 100 μm.

## 3. Results 

### 3.1. Precipitation and Crystalline Formation

**Figure 1:**
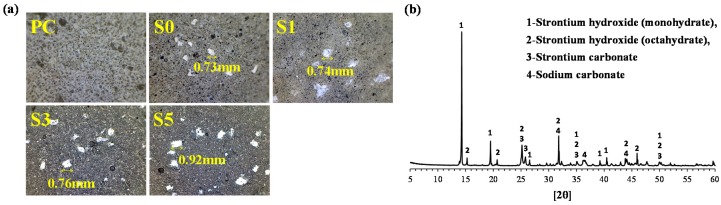
The precipitates formed in the binder matrix incorporating cesium and strontium: (**a**) microscope images and (**b**) X-ray diffraction peak patterns of the precipitates.

Microscope images and the XRD peak patterns of the precipitates in the binder matrix with cesium and strontium are shown in Figure 1. The precipitate in the internal matrices of the alkali-activated cements, including S0, S1, S3, and S5, was white with a diameter of less than 1 mm. The XRD peak patterns revealed that the precipitates mainly consisted of crystalline phases such as strontium hydroxide monohydrate (Sr(OH)_2_·H_2_O) and strontium hydroxide octahydrate (Sr(OH)_2_·8H_2_O). The precipitation of strontium hydroxide in alkali-activated cements was likely to have occurred via reactions with the sodium hydroxide present in the alkali activator, as expressed in Equations (3) and (4):

2NaOH + SrCl_2_·6H_2_O → Sr(OH)_2_·H_2_O + 2NaCl + 5H_2_O, (3)

2NaOH + SrCl_2_·6H_2_O + 2H_2_O → Sr(OH)_2_·8H_2_O + 2NaCl. (4)

Meanwhile, peaks corresponding to strontium carbonate (SrCO_3_) and sodium carbonate (Na_2_CO_3_·H_2_O) were also observed in the XRD peak patterns. These carbonates are artifacts of the reaction with carbon dioxide in the atmosphere, indicating that natural carbonation can also affect the precipitation of strontium ions.

**Figure 2:**
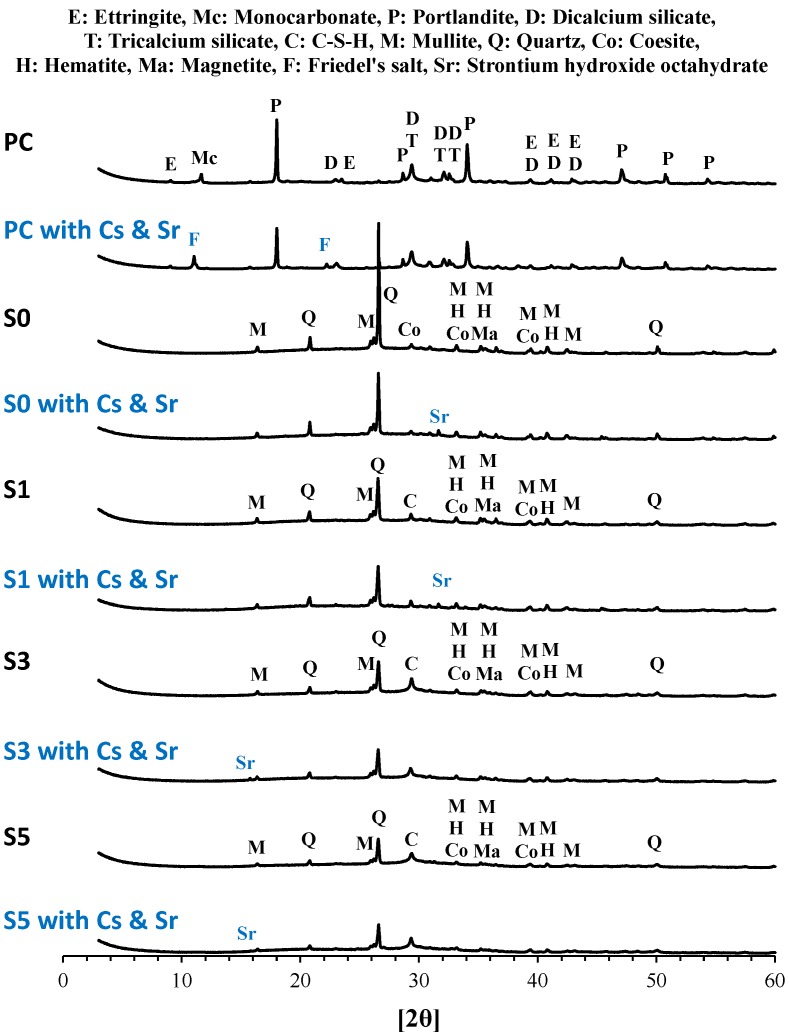
The influence of the incorporated cesium and strontium on the crystalline structures of the binder.

The influence of the incorporated cesium and strontium on the crystalline structures of the binder is shown in Figure 2. In the PC binder, the incorporation of cesium and strontium did not lead to the formation of a crystalline phase bearing both species but in the formation of Friedel’s salt, which is a result of the sulfate ion in monosulfate (AFm) or ettringite (AFt) being replaced by a chloride ion, indicating that the chloride ions from cesium chloride and strontium chloride resulted in the formation of Friedel’s salt [30,31]. In the S0 binder, a fly ash-based binder, the crystalline phases were mullite, quartz, coesite, hematite and magnetite, i.e., unreactive crystalline phases present in the raw fly ash. The peaks corresponding to strontium hydroxide and sodium chloride shown can be identified by the reaction between strontium chloride and sodium hydroxide, as expressed by Equations (3) and (4). The crystalline phases in the fly ash/slag blended binders (S1, S3 and S5) were similar to that of S0, while the peak corresponding to calcium silicate hydrates (C-S-H) increased with the slag content. The incorporation of strontium chloride resulted in the formation of strontium hydroxide, while no cesium-bearing crystalline phase was observed, similar to the results of PC and S0.

### 3.2. Adsorption Kinetics

Determining the adsorption kinetics is a prerequisite to understanding the adsorption mechanism in batch adsorption experiments. Among numerous models of the adsorption kinetics, Lagergren’s pseudo-first-order model [32] and Ho’s pseudo-second-order model [33] are the two that are most widely used. This study investigated the kinetics of cesium and strontium adsorption on a binder using these two models. Lagergren’s pseudo-first-order rate equation and Ho’s pseudo-second-order rate equation are given in a non-linear form, as expressed by Equations (5) and (6), respectively [32,33]:

(5) $$q_{t}=q_{e}\left(1-e^{-k_{1}t}\right),$$

(6) $$q_{t}=\frac{{q_{e}}^{2}k_{2}t}{\left(1+q_{e}k_{2}t\right)}.$$

Here, *q_t_* and *q_e_* are the amounts of cesium and strontium adsorbed on the binder (mg/g) at time t and at equilibrium, respectively; *k*_1_ is the rate constant of the pseudo-first-order model (h^−1^), and *k*_2_ is the rate constant of the pseudo-second-order model (g mg^−1^ h^−1^) [32,33].

**Figure 3:**
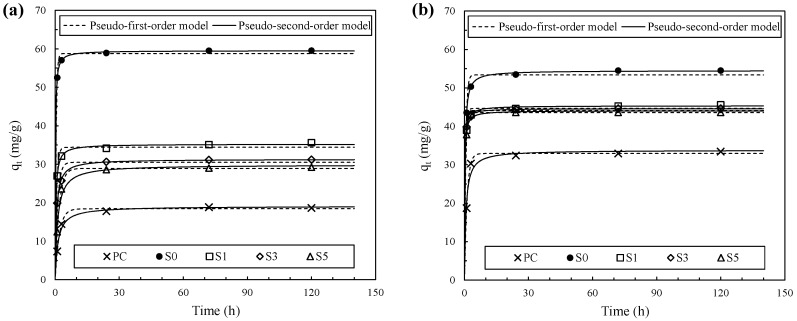
Adsorption capacities and kinetic modelings of (**a**) cesium and (**b**) strontium adsorptions on various binder types.

The cesium and strontium adsorption capacities of the binders are shown in Figure 3 along with the kinetic modeling results. The adsorption of cesium and strontium reached equilibrium within the first 24 h in all binders. The *q_e_* value of cesium after 120 h was 59.56 mg/g in S0, which was the highest, whereas it was 18.65 mg/g in PC, i.e., the lowest. In the fly ash/slag blended binders, an increase in the slag content reduced the *q_e_* value of cesium. The *q_e_* value of strontium was correlated with the binder type, similar to that of cesium, whereas the influence of the binder type on the *q_e_* value of strontium was minor. The *q_e_* value of strontium was highest at 54.52 mg/g in S0 and lowest at 33.44 mg/g in PC. The result of *q_e_* values indicates that physicochemical properties of alkali-activated cements clearly influence the adsorption capacities of cesium and strontium.

It is interesting to note in Figure 3 that the adsorption kinetic of the binders had different compatibility characteristics with the models. The parameters of kinetic models of the cesium and strontium adsorptions on various binder types are listed in Table 3 and Table 4, respectively. The cesium and strontium adsorption kinetics of PC and S5 were in good agreement with the pseudo-first-order model, while those of S0, S1 and S3 matched the pseudo-second-order model well. This can be interpreted as follows: the cesium and strontium adsorption kinetics of S0, S1 and S3 were affected by the complex multistep chemisorption process [34].

**Figure 4:**
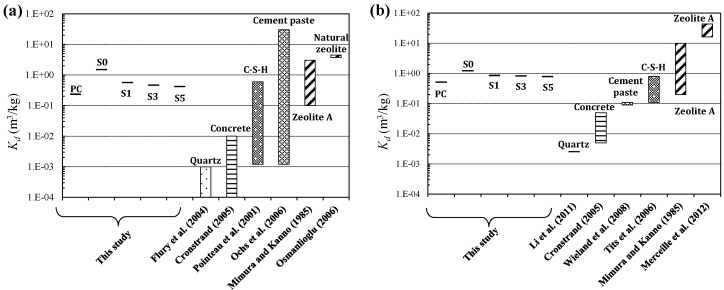
Compilation of *K_d_* values for (**a**) cesium and (**b**) strontium in this study and values from the literature.

The distribution factor *K_d_* of the binders was calculated in each case, as plotted in Figure 4 along with the values obtained in previous studies. The *K_d_* values for cesium and strontium obtained in previous studies were sourced from the data in previous studies [35,36,37,38,39,40,41,42,43,44]. It should be noted that the *K_d_* value can be influenced by the ratio of the adsorbent to the solution, the concentration of the target ion, the ionic strength of the competing ions, the temperature and the pH; hence, comparison of the *K_d_* values can only be done qualitatively.

The *K_d_* value of PC for cesium in this study was 0.23 m^3^/kg, which was slightly higher in comparison with that obtained from the adsorption data for the Portland cement paste (C-S-H) in Pointeau et al. [36] and Ochs et al. [39], while the value in the present study was within the range suggested in previous studies. The *K_d_* values of S1, S3 and S5 for cesium were in the range of 0.41 to 0.55 m^3^/kg, slightly higher than that of PC. Ochs et al. [39] suggested that the adsorption capacity of calcium silicate hydrates (C-S-H) is significantly influenced by the Ca/Si ratio; i.e., a lower ratio tends to yield higher cesium adsorption capacity, showing close agreement with the findings in this study. It is evidenced in previous studies that the Ca/Si ratio of C-S-H hydrates in alkali-activated slag is generally lower than that in Portland cement [45]. It is therefore inferred that the *K_d_* values of alkali-activated slag, being higher than that of PC, were attributed to the lower Ca/Si ratio of the C-S-H hydrates. Similarly, the *K_d_* value decreased with an increase in the slag content of the fly ash/slag blended binder. The *K_d_* value was highest at 1.47 m^3^/kg in the fly ash-based binder S0. In the case of strontium adsorption, the *K_d_* value of PC in this study was 0.50 m^3^/kg, showing a higher value than that of the Portland cement paste (C-S-H) as obtained by Wieland et al. [42]. Meanwhile, the value was within the range corresponding to C-S-H presented in Tits et al. [41]. The *K_d_* value of the fly ash/slag blended binders (S1, S3 and S5) for strontium was 0.77–0.84 m^3^/kg, higher than that of PC. The *K_d_* value of the fly ash-based binder S0 was 1.20 m^3^/kg, showing the highest value among all binders.

### 3.3. Characteristics of Gel in Alkali-Activated Cements

The composition of binder gel in alkali-activated cements is highly dependent on the precursor used for the synthesis and determines its chemical and mechanical properties. Firstly, the binder gel in alkali-activated slag predominantly consists of amorphous C-S-H as similar with that in Portland cement, while its Ca/Si ratio is lower and includes occasions of Al substitution for tetrahedral Si in the chain structure of C-S-H [45]. The Na supplied with the activator also forms a structural part in the C-S-H gel by acting as a charge balancer for Al; therefore, it is also referred to as C-(N)-A-S-H [46]. On the other hand, alkali-activated fly ash comprises of amorphous aluminosilicate gel in which tetrahedral Si and Al are interconnected in a 3D framework, forming a polymer structure [17,27]. In an alkali-activated fly ash/slag blended binder, these two gels coexist with varying composition depending on the ratio of fly ash and slag, and higher degree of crosslinking in C-(N)-A-S-H gel in comparison with that in alkali-activated slag is also observed [16,46].

**Figure 5:**
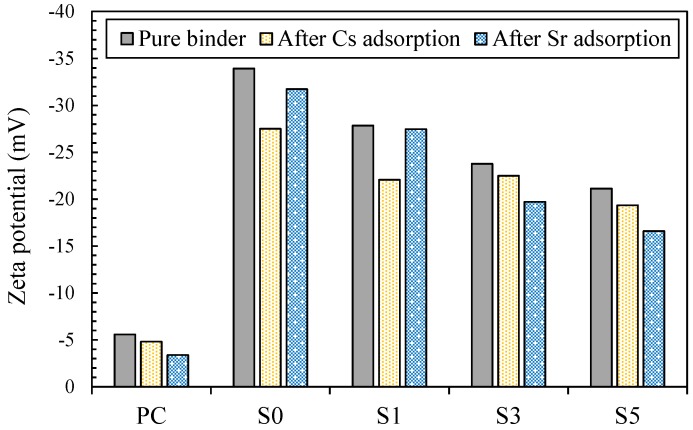
Zeta potential values of powdered binders before and after the adsorption tests.

The zeta potential values of the powdered binders before and after the adsorption tests are shown in Figure 5. The zeta potential value was −5.6 mV and highest in PC, while the value became more negative in the fly ash/slag blended binder as the slag content decreased. The zeta potential value of S0 was −33.9 mV, indicating that the surface of S0 was highly negatively charged. This may be attributed to the alkaline activation product of fly ash which has much less Ca, therefore tending to a more negative charge on the surface. It was reported in previous studies that a negatively charged zeta potential of a particle is more favored in terms of the cation adsorption capacity (e.g., [47,48,49]). The zeta potential values of the powdered pure binders generally showed a high correlation with the *q_e_* values for cesium and strontium. Meanwhile, the zeta potential values of the binders after the adsorption of cesium or strontium became more positive. The results overall indicate that the aluminosilicate gels in alkali-activated fly ash generally had a highly negatively charged surface, which, in turn, was more effective for adsorbing cesium and strontium by electrostatic interactions and the chemisorption mechanism.

**Figure 6:**
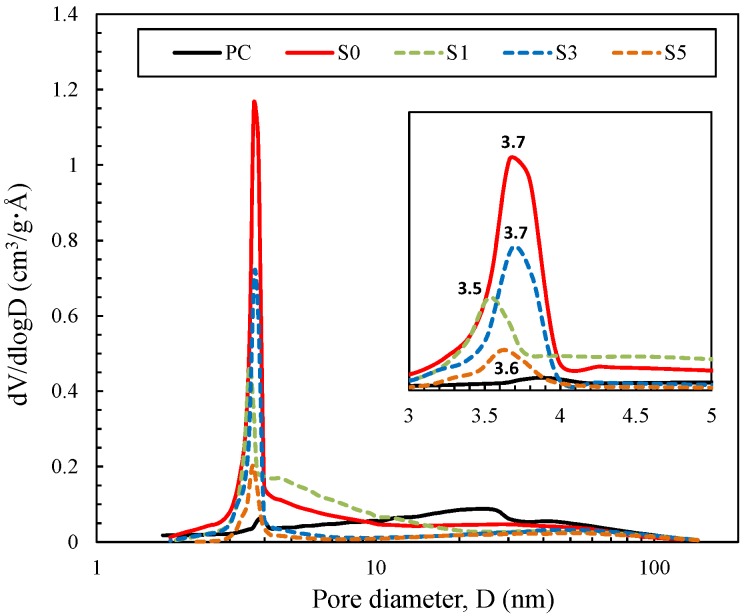
Pore size distribution of powdered binders measured by a BJH N_2_ desorption method.

The pore size distributions of the powdered binders as measured by the BJH N_2_ desorption method are shown in Figure 6, and the pore characteristics of the powdered binders, as measured by the BET/BJH methods, are summarized in Table 5. These results show that pores with a diameter of 3.6 ± 0.1 nm were present in the alkali-activated cements (S0, S1, S3 and S5), while those were not significantly present in PC. The surface area and pore volume decreased in the fly ash/slag blended binder as the slag content increased. S0 had the highest surface area and micropore volume, indicating that these pore characteristics of S0 were the most adequate in terms of adsorption of cesium and strontium. Accordingly, this also implies the fly ash-based binder is likely to be more effective and efficient in terms of chemical barriers for preventing the release of cesium and strontium.

## 4. Discussion

Previous studies dealing with the leaching behavior of cesium and strontium from alkali-activated cements commonly reported significantly higher leaching resistance in comparison with Portland cement-based matrix [21,22,24,25]. A recent study conducted by the authors showed that the higher leaching resistance of cesium or strontium was found to be attributed to the physical barrier effect of alkali-activated fly ash to some extent, while the chemical aspect may govern the immobilization capacity [24]. In the present study, the performance of the fly ash-based binder (S0) during the immobilization of cesium was the most outstanding, while that of the fly ash/slag blended binder was middling among the three binder systems. XRD and microscope image analyses suggested that the incorporation of cesium in the binders used in this study did not result in any crystalline or precipitate formation. This observation differs significantly from the reaction of strontium expressed as Equations (3) and (4). The solubility of cesium hydroxide is the most likely cause, as it occurs at 300 g/100 mL (30 °C), much higher as compared to strontium hydroxide (1.77 g/100 mL at 20 °C), calcium hydroxide (0.173 g/100 mL at 20 °C) and sodium hydroxide (111 g/100 mL at 20 °C). In other words, an alkali activator with a high pH level can be beneficial in terms of strontium precipitation, while it remains uncorrelated with the precipitation of cesium.

The batch adsorption test results showed that the fly ash/slag blended binder with a lower content of slag had a higher capacity of cesium adsorption. This implies that the adsorption capacity of the alkali-activated cement was strongly influenced by the presence and extent of the aluminosilicate gel (PC < S5 < S3 < S1 < S0). As discussed in previous studies, calcium silicate hydrate and aluminosilicate gel coexist in the matrix of the fly ash/slag blended binder, and the amounts of these two gel types are determined by the slag content [26,46]. The immobilization capacity of the slag-blended binder system is therefore governed by the extent of the aluminosilicate gel, which has an effective capacity for cesium adsorption. Another interesting phenomenon important to note is the compatibility of the binder system with a high adsorption capacity with a pseudo-second-order model as evidenced by adsorption kinetic modeling. This can be interpreted to conclude that the immobilization mechanism of cesium in alkali-activated fly ash is governed by the cation exchange mechanism [50]. Furthermore, this phenomenon was attributed to the highly negatively charged surface, the higher surface area, and the micropore volume of the alkali-activated fly ash, in comparison with those of other binders.

The last point remaining to be discussed is the possibility of cesium being incorporated into an amorphous aluminosilicate gel. Bell et al. [51] explored the analogue of cesium aluminosilicate gel (Cs_2_O·Al_2_O_3_·4SiO_2_·*x*H_2_O, with *x* ~ 11) by means of the atomic pair distribution function method. An XRD peak corresponding to well-crystallized pollucite (CsAlSi_2_O_6_) was observed in the cesium aluminosilicate binder heated at a temperature of 1000 °C above, and the sample cured at an ambient temperature displayed structural ordering similar to that of pollucite with a smaller length scale [51]. They also reported that the hydrated Cs^+^ ion was an integral part of the cesium aluminosilicate structure [51]. It is suggested that the alkali-activated fly ash is an effective medium for immobilizing cesium, as its aluminosilicate gel interacts with cesium via incorporation into the amorphous gel and chemisorption with a negatively charged surface.

The most dissimilar aspect of strontium, in light of their immobilization mechanism in alkali-activated cements, was the formation of crystalline precipitates. The formation of the crystalline strontium precipitates of strontium hydroxide in fly ash-based binder and fly ash/slag blended binder were evidenced by microscope imagery and an XRD analysis. The batch adsorption test result showed that the incorporation of slag did not influence the strontium adsorption capacity of the alkali-activated cement. Moreover, the adsorption capacity of Portland cement for strontium was greater than that of cesium by a factor of two. Considering the considerable influence of the binder type on the adsorption capacity for cesium, this observation suggests that the formation of calcium silicate hydrate by the alkali-activation of slag contributed to strontium adsorption to some extent. In particular, the calcium silicate hydrate formed by the alkali-activation of the slag inherits lower Ca/Si than that of Portland cement, indicating that the calcium silicate hydrate in alkali-activated slag is more effective for strontium adsorption than Portland cement. In conclusion, the alkali-activated fly ash is an effective medium for immobilizing strontium by a mechanism similar to that of cesium.

## 5. Conclusions

The present study investigated the retention mechanisms of cesium and strontium in alkali-activated cements. Possible retention mechanisms, including adsorption and precipitation, were examined in light of the chemical interactions. The following conclusions can be drawn considering the results of this work:

(1) The incorporation of cesium in all binders did not result in the formation of a new crystal or change in the crystalline phases, whereas that of strontium resulted in precipitation of strontium hydroxide and strontium carbonate. The higher frequency of strontium precipitation in alkali-activated cements is attributed to the use of an alkali-activating solution with a high pH.
(2) Batch adsorption test results showed that the cesium and strontium adsorption of the binders were in the following order: PC < S5 < S3 < S1 < S0. The adsorption kinetics of PC and S5, with relatively low adsorption capacities for cesium and strontium, were compatible with Lagergren’s pseudo-first-order model, while those of S3, S1 and S0, with relatively higher adsorption capacities, were compatible with Ho’s pseudo-second-order model. These results indicate that the cesium and strontium adsorption kinetics of S3, S1 and S0 were affected by the complex multistep chemisorption process.
(3) The zeta potential and BET surface area values of binders showed a high correlation with the adsorption capacities for cesium and strontium. Alkali-activated fly ash had a highly negatively charged surface as well as the highest surface area and mesopore volume, indicating that these characteristics of aluminosilicate gel facilitated the more effective immobilization of cesium and strontium through electrostatic interactions and the chemisorption mechanism in comparison with the calcium silicate hydrate gel.

## Figures and Tables

**Table 1:** Chemical compositions of the raw materials used in this study.

(wt %)	Fly Ash	Slag	Portland Cement
CaO	5.3	47.7	62.5
SiO_2_	51.5	32.4	21.0
Al_2_O_3_	22.0	11.5	5.9
Fe_2_O_3_	10.8	0.6	3.2
MgO	2.0	3.0	2.8
K_2_O	1.2	0.5	0.9
TiO_2_	1.6	0.5	-
SO_3_	0.7	2.7	2.1

**Table 2:** Mix design of the samples.

Sample Code	Mix Design (Relative Weight Proportion)	Initial Curing (for 24 h)
PC	Portland cement (1.0) + Water (0.5)	20 °C
S0	Fly ash (1.0) + 9 M NaOH (0.25) + Sodium silicate (0.25)	60 °C
S1	Fly ash (0.9) + Slag (0.1) + 4 M NaOH (0.33) + Sodium silicate (0.17)	20 °C
S3	Fly ash (0.7) + Slag (0.3) + 4 M NaOH (0.33) + Sodium silicate (0.17)	20 °C
S5	Fly ash (0.5) + Slag (0.3) + 4 M NaOH (0.33) + Sodium silicate (0.17)	20 °C

**Table 3:** Parameters of kinetic models of cesium adsorptions on various binder types.

Binder	Experimental	Pseudo-First-Order Model			Pseudo-Second-Order Model		
	$q_{e}$ (mg/g)	$k_{1}$ (h^−1^)	$q_{e}$ (mg/g)	*R*^2^	$k_{2}$ (g mg^−1^ h^−1^)	$q_{e}$ (mg/g)	*R*^2^
PC	18.65	0.510	18.44	0.994	0.040	19.09	0.976
S0	59.57	2.230	58.80	0.888	0.127	59.52	0.997
S1	35.61	1.492	34.41	0.887	0.093	35.23	0.988
S3	31.21	0.861	30.50	0.911	0.054	31.32	0.998
S5	29.22	0.563	28.94	0.999	0.029	29.87	0.970

**Table 4:** Parameters of kinetic models of strontium adsorptions on various binder types.

Binder	Experimental	Pseudo-First-Order Model			Pseudo-Second-Order Model		
	$q_{e}$ (mg/g)	$k_{1}$ (h^−1^)	$q_{e}$ (mg/g)	*R*^2^	$k_{2}$ (g mg^−1^ h^−1^)	$q_{e}$ (mg/g)	*R*^2^
PC	33.44	0.843	32.92	0.996	0.044	33.80	0.935
S0	54.52	1.662	53.38	0.887	0.073	54.49	0.997
S1	45.59	2.048	44.67	0.872	0.135	45.33	0.991
S3	44.65	2.119	44.10	0.836	0.183	44.61	0.994
S5	43.64	2.030	43.56	0.998	0.154	44.03	0.914

**Table 5:** Pore characteristics of powdered binders.

Binder	PC	S0	S1	S3	S5
BET surface area (m^2^/g)	36.2	77.6	55.6	37.8	12.72
BJH cumulative volume of pores (cm^3^/g)	0.089	0.138	0.116	0.071	0.037
BJH average pore diameter (nm)	9.8	5.2	5.9	5.4	8.0
